# MRI features of parenchymal neuro-Behçet’s disease: a systematic review and meta-analysis

**DOI:** 10.3389/fneur.2026.1818678

**Published:** 2026-08-19

**Authors:** Hind Alnajashi, Hussain Ali J. Almohammed, Ahmed Salah Morad, Bushra Wadi Bin Saddiq, Kawthar Faisal Kushara, Tanveer Nidal Khan, Wejdan Ahmed Aldawsari, Hadeel Hesham Buali, Fatimah Ibrahim Almuhaysin, Mayar Ahmed Gasim, Hams Akram Alharbi, Abdullah Ahmed Albesher

**Affiliations:** 1 Department of Neurology, Faculty of Medicine, King Abdulaziz University, Jeddah, Saudi Arabia; 2 College of Medicine, King Faisal University, Al Ahsa, Saudi Arabia; 3 General Medicine Practice, Batterjee Medical College, Jeddah, Saudi Arabia; 4 College of Medicine, King Abdulaziz University, Jeddah, Saudi Arabia; 5 College of Medicine, Tabuk University, Tabuk, Saudi Arabia

**Keywords:** anatomical distribution, brain stem atrophy, disease severity, enhancing lesion, magnetic resonance imaging, neuro-Behçet’s disease

## Abstract

**Introduction:**

Magnetic resonance imaging (MRI) is essential for diagnosing neuro-Behçet’s disease (NBD). The classic MRI sign typically results from parenchymal NBD lesions in the thalamus, midbrain, and the internal capsule. It may also affect the spinal cord, bilateral basal ganglia, pons, and white matter. Despite its importance, a comprehensive quantitative synthesis of MRI characteristics differentiating NBD subtypes is still needed. This study aimed to compare specific MRI findings, such as brainstem atrophy, enhancing lesions, and anatomical distribution, in acute and chronic progressive parenchymal NBD, with potential implications for prognosis and long-term disease management.

**Methods:**

This systematic review and meta-analysis was conducted according to the PRISMA 2020 guidelines. PubMed, Web of Science, and Scopus were searched from inception to August 2025. Retrospective studies assessing the MRI findings of parenchymal NBD were included.

**Results:**

Seven retrospective studies (total *n* = 327) with parenchymal NBD met the inclusion criteria. The characteristic lesions showed that brainstem atrophy was significantly less in the acute NBD group than in the chronic progressive NBD group (OR = 0.03, 95% CI [0.01, 0.11], *p* < 0.00001). Contrast-enhancing lesions were significantly more frequent in acute parenchymal NBD, consistent with active inflammatory disease (OR = 2.58, 95% CI [1.04, 6.40], *p* = 0.04). However, the anatomical distribution of MRI lesions does not differ significantly between acute and chronic progressive NBD. No significant difference was observed between the mild-to-moderate and severe groups in the brainstem (OR = 0.42, *p* = 0.22) and thalamus (OR = 0.31, *p* = 0.12).

**Conclusion:**

Our analysis provides quantitative evidence for distinct MRI characteristics, such as differential brainstem atrophy and enhanced lesion incidence, which may inform NBD subtyping and have implications for prognosis and long-term disease management. However, this meta-analysis is limited by the retrospective nature of the included studies and their potential heterogeneity, necessitating further prospective research to confirm these findings.

**Systematic review registration:**

PROSPERO, CRD420251178483.

## 1 Introduction

Behçet’s disease, also known as the Silk Road disease, is a systemic inflammatory vascular disorder with autoinflammatory characteristics, although its precise origin has not been determined (1). The clinical spectrum of Behçet’s is characterized by multisystem involvement with episodic exacerbations and remissions. The principal features include recurrent mucocutaneous ulcerations affecting the oral and genital mucosa, ocular inflammation manifesting as uveitis, and musculoskeletal complaints involving the major joints. Severe complications may arise from vasculitic processes affecting both the venous and arterial systems, with potential gastrointestinal and central nervous system involvement (2).

The geographic distribution of Behçet’s syndrome demonstrates marked regional variation, with a relatively low occurrence in North American and Western European populations. In contrast, a substantially elevated prevalence is observed in countries positioned along the historic Silk Road trading corridor, which historically linked East Asian territories to the Mediterranean regions. Turkey exhibits the highest incidence rates globally, while other nations, including Saudi Arabia, Japan, Korea, China and Iran, report considerable disease prevalence (3). In addition to exhibiting a higher prevalence of anxiety, depression, and cognitive impairments than the general population, patients with Behçet’s disease commonly experience substantial deterioration in their physical capacity, psychological wellness, and social engagement (4).

The neurological form of Behçet’s disease, referred to as neuro-Behçet’s disease (NBD), is a particularly severe variant of Behçet’s disease, characterized by rapid progression and associated with significant morbidity and mortality rates (5). Although most studies report CNS involvement in 15–25% of patients with Behçet’s disease, another study demonstrated a considerably higher prevalence, with neurological manifestations occurring in more than 50% of 41 patients (6). NBD can be divided into two main categories: those affecting brain tissue (parenchymal) and those involving blood vessels (nonparenchymal), with an additional mixed category for patients showing characteristics of both types. Notable differences in clinical features were observed between the groups. Parenchymal patients typically exhibit visual problems, motor dysfunction, and cranial nerve deficits, whereas nonparenchymal patients show severe headaches, optic nerve swelling, and increased intracranial pressure (7). The lack of specific biomarkers, reliance on non-specific CSF findings, and MRI that can easily mimic other neurological conditions such as multiple sclerosis, and highly variable clinical presentations ranging from acute meningoencephalitis to chronic progressive neurological symptoms make diagnosing NBD difficult (8).

When clinical symptoms indicate NBD, it is essential to use contrast-enhanced brain magnetic resonance imaging along with magnetic resonance venography to determine the specific type of neurological involvement (8, 9). The classic cascade sign on MRI typically results from parenchymal lesions at the mesodiencephalic junction, involving the thalamus, midbrain, and internal capsule. It may also affect the spinal cord, bilateral basal ganglia, pons, and subcortical white matter. During the acute phase, these lesions show patchy enhancement, appearing T1 hypointense and T2 hyperintense, with accompanying vasogenic edema and a potential mass effect (10).

There is significant variability in the diagnostic techniques used across various studies, particularly concerning neuroimaging methods, such as differences in MRI protocols and scan interpretations. This inconsistency complicates the standardization of imaging results and makes it difficult to draw consistent conclusions regarding the typical imaging patterns in NBD (7, 9, 11). Although MRI is a crucial diagnostic tool for NBD, studies on its imaging characteristics remain inconsistent and challenging to interpret. Existing research employs different MRI techniques, applies varying diagnostic criteria, and reports findings in non-uniform ways. This gap has significant clinical implications, as inconsistent interpretations of imaging findings lead to diagnostic delays, frequent misdiagnoses, and complications in treatment decisions. This study aimed to identify the MRI findings reported in patients with NBD.

## 2 Methodology

### 2.1 Protocol approval, registration, and patient consent

This systematic review followed the Preferred Reporting Items for Systematic Reviews and Meta-analyses (PRISMA) 2020 guidelines (12). The protocol for this study was registered with PROSPERO (registration number: CRD420251178483). This study did not require ethical approval or patient permission because of its nature of the study.

### 2.2 Search strategy

The electronic databases PubMed, Web of Science, and Scopus were systematically searched to extract all relevant articles published until August 2025. A combination of Medical Subject Heading (MeSH) terms, free-text fields, and Boolean operators was used to optimize the search results. The keywords included in the search strategy were following: (Behcet Syndrome OR Behçet OR Neuro Behcet OR Neuro-Behçet’s disease OR NBD) AND (Magnetic Resonance Imaging OR MRI OR magnetic resonance OR MR imaging OR diffusion-weighted OR DWI OR FLAIR OR SWI OR contrast-enhanced) AND (parenchymal OR parenchymal NBD OR nonparenchymal OR nonparenchymal NBD). The complete search strings are provided in Supplementary Table 1.

### 2.3 Study selection criteria

Studies were eligible for inclusion if they were published in English and included patients aged ≥ 18 years with a confirmed diagnosis of NBD. Patients had to meet the criteria of the International Study Group for BD, old or new, and had to present with neurological manifestations in the included studies (13, 14). NBD was defined as neurological manifestations in the absence of an alternative condition that better explained these symptoms. Studies have used MRI to identify lesions between acute parenchymal NBD and parenchymal chronic progressive NBD. Patients were described as having acute NBD when they displayed acute focal neurological symptoms. Patients were classified as having the chronic progressive type if they displayed continuous worsening of neurological symptoms or signs over months or years. Studies have compared MRI findings in mild-to-moderate and severe NBD. The modified Rankin Scale (mRS) was used to assess NBD severity in the included studies. The mRS scores categorized patients with NBD into mild-to-moderate (scores 0–3) or severe (scores 4–6) groups. Only retrospective studies were included in this review because they did allow differentiation between acute and chronic progressive disease courses or assessment of temporal MRI characteristics. In contrast, case reports, cross-sectional studies, case–control studies, opinion papers and editorials, review articles, and studies that did not report outcomes of interest or possible high heterogeneity for analysis were excluded from the study.

### 2.4 Screening

Four reviewers (KK, FA, HA, and MG) screened all extracted articles. Each reviewer independently performed an initial screening by title and abstract using Rayyan software (15). Articles that met the inclusion criteria were subjected to full-text screening by a single reviewer. Any disagreements were resolved through discussion.

### 2.5 Data extraction and risk of bias assessment

The data retrieved from the studies included the following details: study characteristics (author’s last name, year of publication, study design, country of origin, period, diagnostic criteria, time to NBD, MRI imaging and follow-up). Baseline characteristics of the included studies (number of patients, age of onset (years), HLA-B51, and clinical characteristics). One reviewer (HAJA) created a datasheet, which was then evaluated by another reviewer (AM). Two reviewers (HAJA and BB) independently extracted data. Disagreements were resolved by consensus or consultation with a third reviewer (KK). The obtained data were double-checked to eliminate any duplication. The risk of bias was assessed at the outcome level of each study using the Newcastle–Ottawa Scale (NOS) for retrospective studies (16).

### 2.6 Outcome measures

The primary outcomes of interest were to identify the location of lesions observed on MRI (brainstem, white matter, basal ganglia, cerebellum, spinal cord, and thalamus) in parenchymal NBD, compare lesion distribution between acute and chronic progressive parenchymal NBD, and characterize MRI features. In addition, the lesions were compared according to disease severity. The secondary outcome was the resolution of brain lesions on follow-up MRI compared with persistent lesions.

### 2.7 Data analysis

This meta-analysis was conducted to utilize the findings from the studies included in the search to determine the location of lesions seen on MRI in patients with parenchymal NBD. Statistical analysis was conducted using RevMan Manager version 5.0 software, which aided in pooling the study results and evaluating the overall effects (17, 18). Heterogeneity across studies was examined using the Cochrane-based *Q* statistic and *I*^2^ test, which determined the type of model to be used in the analyses. A random-effects model was applied when substantial heterogeneity was present (*p* < 0.05, *I*^2^ > 50%). A fixed-effects model was used when heterogeneity was minimal (*I*^2^ < 50%, *p* > 0.05). Standardized mean differences (SMD) for continuous outcomes and odds ratios (OR) for dichotomous variables were used to express the location of the lesions seen on MRI; each had a 95% confidence interval (CI). Statistical significance was set at *p* < 0.05.

## 3 Results

### 3.1 Search results

**Figure 1:**
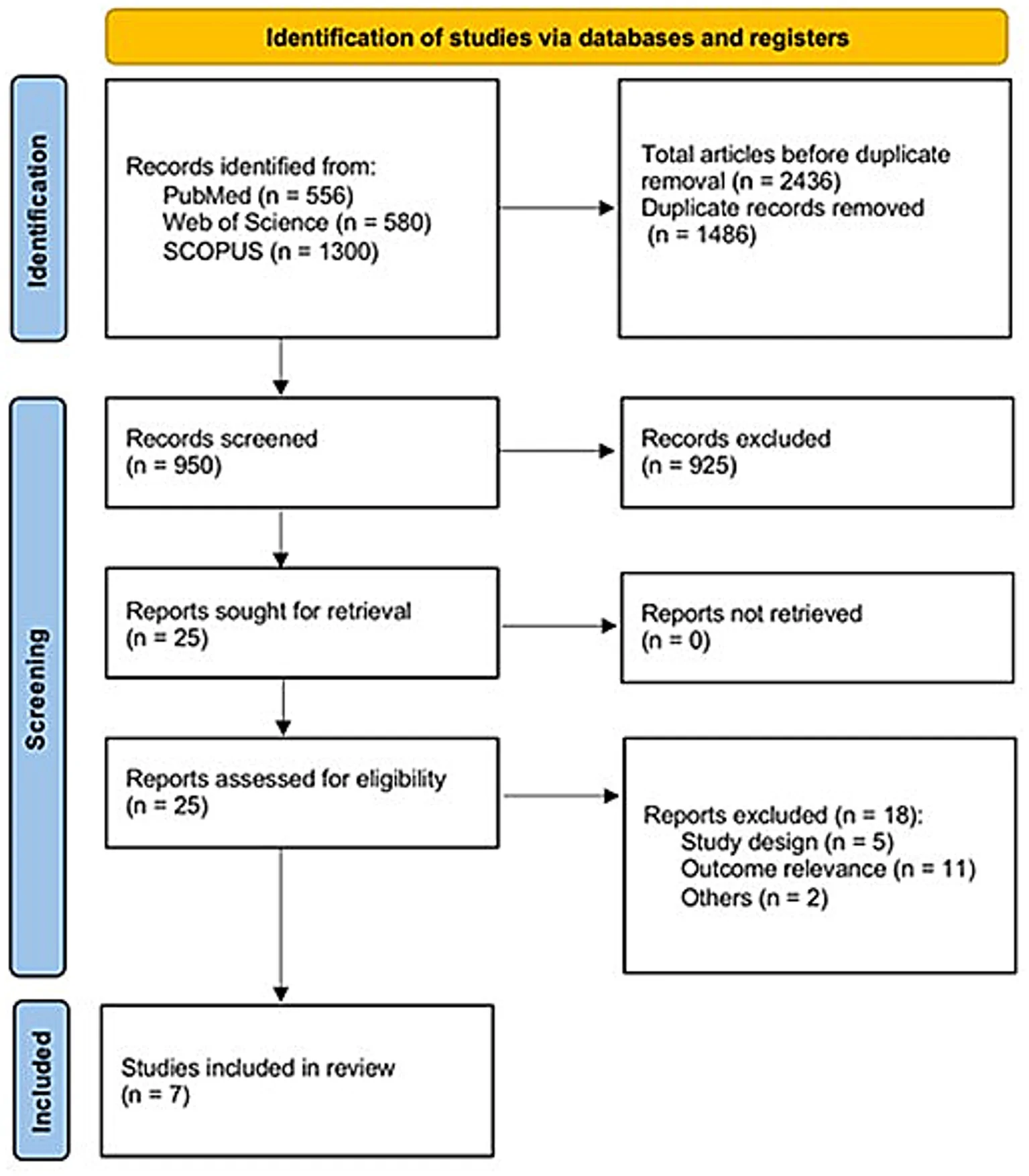
PRISMA flowchart.

Figure 1 illustrates the PRISMA flowchart depicting the steps of study inclusion in this review. Using our search strategy, we retrieved 2,436 studies from PubMed, Scopus, and Web of Science databases. After the removal of 1,486 duplicates, 950 records were retained for screening. Of these, 925 records were excluded because they were irrelevant to the research question. The remaining 25 full-text articles were assessed for eligibility. After a full-text review, 18 studies were excluded. Finally, seven studies were included in this systematic review and meta-analysis (11, 19–24).

### 3.2 Study characteristics of NBD

Table 1 summarizes the characteristics of the included studies in this review. The studies were conducted in multiple countries. Two studies originated in Turkey (19, 22), while each of the remaining studies had a different country of origin, including Lebanon (11), South Korea (20), Iran (21), France (23) and Japan (24). The studies spanned different periods of time; the longest study period was 36 years by Noel in 2014 (23), while the shortest period was 7 years by both Sorgun in 2018 (19) and Haghighi in 2010 (21). All studies used the ISG-BD diagnostic criteria in their protocols, except for Ideguchi 2010 (24), which used the Japanese criteria revised in 1987 for the diagnosis of Behçet’s.

**Table 1:** Study characteristics.

Study ID	Country	Study design	Study period	Diagnosis criteria	Follow up
Sorgun 2018 (19)	Turkey	Retrospective	January 2007–June 2014	ISG-BD	N/A
Chamoun 2025 (11)	Lebanon	Retrospective	January 2000–January 2021	ISG-BD	N/A
Kim 2019 (20)	South Korea	Retrospective	January 2000–December 2017	ISG-BD	N/A
Haghighi 2010 (21)	Iran	Retrospective	January 2002–January 2009	ISG-BD	N/A
Demir 2003 (22)	Turkey	Retrospective	N/A	ISG-BD	8.5 days–46 months
Noel 2014 (23)	France	Retrospective	1974–2010	ISG-BD	73 (59–102) months
Ideguchi 2010 (24)	Japan	Retrospective	July 1991–December 2007	Japanese criteria revised in 1987 for diagnosis BD	7.5 ± 6.7 years

ISG-BD, International Study Group for Behçet’s Disease.

### 3.3 Baseline characteristics of parenchymal NBD

Table 2 shows the baseline characteristics of the participants with parenchymal NBD in the included studies. Collectively, these studies included 327 participants, including 161 patients with acute NB and 67 with chronic progressive NB. The remaining 98 patients were not classified as having acute or chronic progressive NB. The mean age of patients when first diagnosed with BD was 32.8 ± 11.5 years, and the mean age of patients who developed NBD was 38.1 ± 10.8 years. Of the 327 patients, 241 underwent HLA genetic testing. Approximately one-third of these patients had the HLA-B51 antigen (*n* = 84, 34%).

**Table 2:** Baseline characteristics of parenchymal NBD.

Study ID	Number of participants (*n*)			Mean age at BD, years		Mean age at NBD, years		HLA-B51, *n*; %	
	Acute NB	Chronic progressive NB	Total	Acute NB	Chronic progressive NB	Acute NB	Chronic progressive NB	Acute NB	Chronic progressive NB
Kim 2019 (20)	N/A		98	28.2 ± 10.1		37.9 ± 10.6		19; 46.3%	
Haghighi 2010 (21)	9	8	17	36.4 ± 8.1		N/A		N/A	
Demir 2003 (22)	36	7	43	35.4 ± 8.9		N/A		N/A	
Noel 2014 (23)	78	37	115	31.4 ± 12.8	39.4 ± 11.9	34.7 ± 8.2	42.9 ± 13.0	33; 52%	13; 45%
Ideguchi 2010 (24)	38	15	54	34.8 ± 10.7	38.2 ± 10.9	38.1 ± 9.9	44.4 ± 13.6	12/21; 57%	5/7; 71%
Total: 5 studies	Total: 161	Total: 67	Total: 327	Total: 32.8 ± 11.5		Total: 38.1 ± 10.8		Total: 82; 34%	

BD, Behçet’s disease; NBD, Neuro-Behçet’s Disease, HLA-B51; Human Leukocyte Antigen B51.

### 3.4 Clinical characteristics of parenchymal NBD

Supplementary Table 2 presents the clinical data of patients with parenchymal NBD in the studies included. Oral aphthous ulcers have been reported in up to 100% of patients across multiple cohorts (20, 23, 24), highlighting them as the most common systemic presentation in all patients. Genital ulcerations and ocular manifestations followed closely, involving over two-thirds of the participants in most cohorts. Headache is a more common neurological manifestation of acute NB than that of chronic progressive NB (23, 24). In contrast, more severe neurological manifestations, such as ataxia, sphincter dysfunction, and sensory affection, were more common in chronic progressive NB, suggesting a more severe neurological burden in this group.

### 3.5 Baseline characteristics, clinical manifestations of NBD based on disease severity

Supplementary Tables 3, 4 illustrate the baseline characteristics and clinical manifestations of patients with NBD based on disease severity in the included studies. In both studies, patients with severe NBD tended to be older and had an older age of NBD onset than those with mild-to-moderate NBD. Oral ulcers were present in almost all patients included in the study, followed by genital ulcers, uveitis, and, less commonly, GI involvement. As for the neurologic manifestations reported by Chamoun et al. in 2025 (11), headache was more prevalent in patients with mild-to-moderate NBD (*n* = 19; 95%) than in patients with the severe type (*n* = 5; 71.4%). Similarly, for dizziness and weakness, the same trend was observed, with a higher incidence in the mild-to-moderate cohort.

### 3.6 Quality assessment

**Figure 2:**
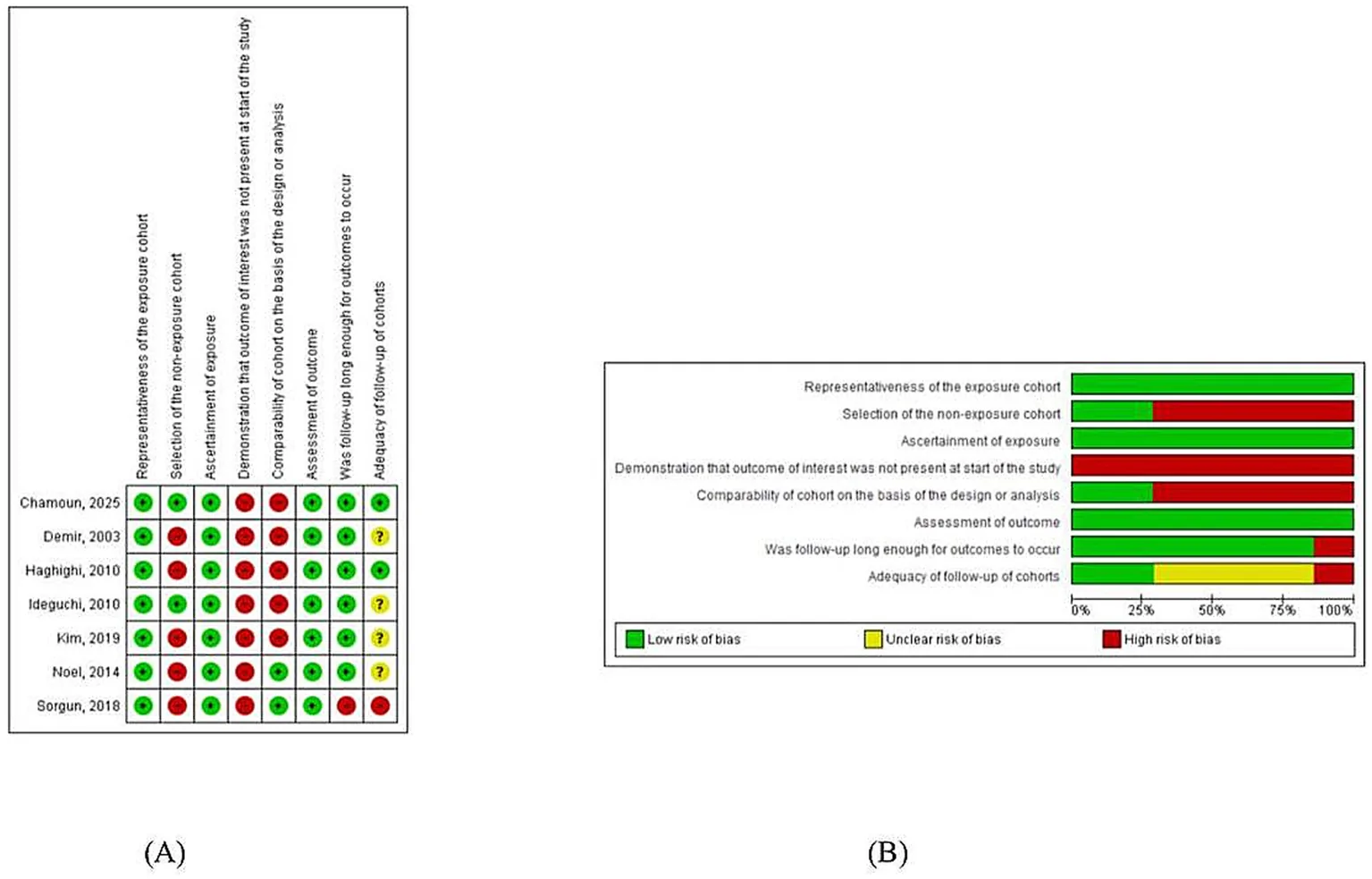
**(A)** Summary of the NOS score result of the included studies. **(B)** Quality assessment of included studies.

The risk of bias in the included studies was evaluated using the NOS, which covers three main domains: selection, comparability, and outcome. Overall, most of the included cohort studies showed a moderate risk of bias, with total scores ranging from four to six stars. A few studies had higher concerns, such as Sorgun et al. (19), which scored three stars and was therefore considered to be at a high risk of bias. Only one study Chamoun et al. (11) demonstrated a relatively strong methodology, with a total score of six stars. The most frequent issues across studies were the absence of a non-exposed comparison group, lack of confirmation that the outcome was not present at baseline and limited or no adjustment for potential confounders in the analysis. Moreover, the studies were retrospective, which may have introduced recall and selection biases. None of the studies reached the low-risk category (seven or nine stars). The risk of bias summary and graphs are shown in Figure 2. Detailed domain-level assessments of the included studies are provided in Supplementary Table 5.

### 3.7 Outcomes

#### 3.7.1 Summary of the outcomes

Table 3 summarizes the outcomes of the meta-analysis of the included studies.

**Table 3:** Summary of outcomes.

Outcomes			Number of studies	Number of patients (A/CP), (M/S)	Heterogeneity		Model used (fixed/random)	Effect size		
					*p*	*I*^2^ (%)		OR	95% CI	*p*
Acute vs chronic progressive	Site of lesion on MRI	Brain stem	3	172/69	0.37	0	Fixed	1.19	[0.66, 2.15]	0.56
		White matter	3	134/56	0.36	3	Fixed	0.92	[0.45, 1.89]	0.82
		Basal ganglia	2	98/39	0.67	0	Fixed	1.86	[0.77, 4.44]	0.17
		Cerebellum	2	98/39	0.97	0	Fixed	1.11	[0.27, 4.50]	0.89
		Spinal cord	2	98/39	0.1	67	Fixed	1.64	[0.44, 6.09]	0.46
		Thalamus	2	98/39	0.41	0	Fixed	1.93	[0.69, 5.42]	0.21
	Brain stem atrophy		2	72/32	0.25	24	Fixed	0.03	[0.01, 0.11]	<0.00001*
	Enhancing lesion		2	72/32	0.29	11	Fixed	2.58	[1.04, 6.40]	0.04*
Mild to moderate vs severe	Site of lesion on MRI	Brain stem	2	76/11	0.18	44	Fixed	0.42	[0.10, 1.68]	0.22
		Thalamus	2	76/11	0.63	0	Fixed	0.31	[0.07, 1.36]	0.12

*p-value is significant. A; Acute, CP; Chronic Progressive, M; Mild to Moderate, S; Severe, OR; Odds ratio; CI; Confidence interval.

#### 3.7.2 Site of lesions seen on MRI imaging of parenchymal NBD

**Figure 3:**
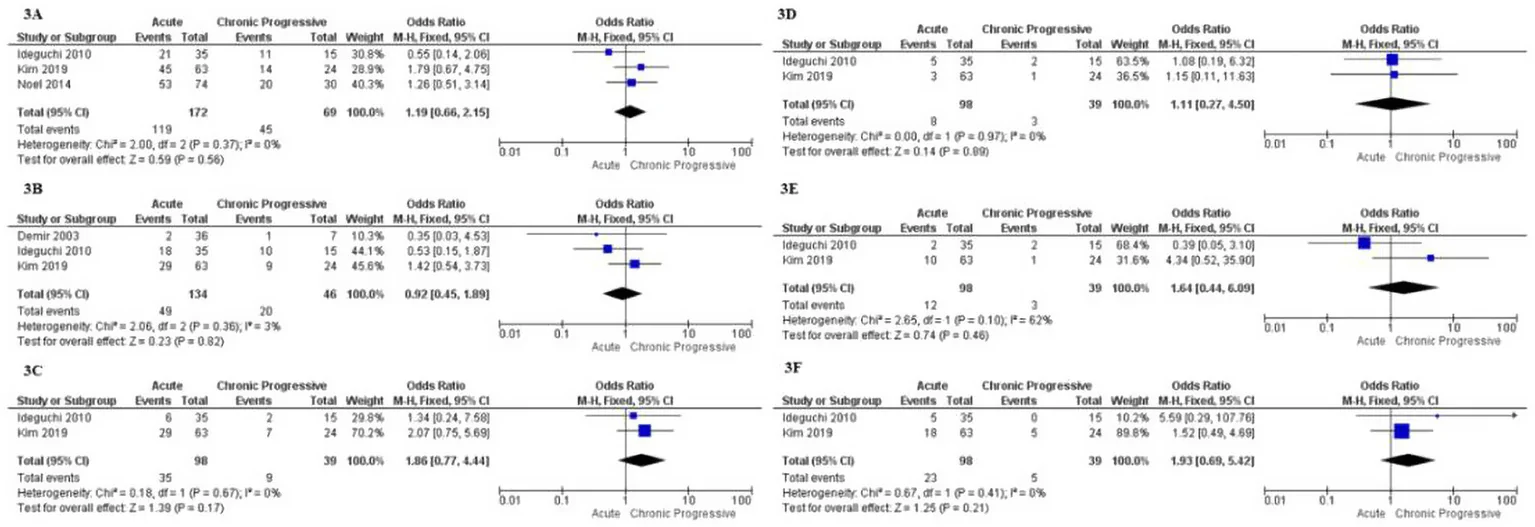
OR of site of lesions seen on MRI imaging between acute and chronic progressive NBD: **(A)** Brainstem, **(B)** White matter, **(C)** Basal ganglia, **(D)** Cerebellum, **(E)** Spinal cord, **(F)** Thalamus.

Figure 3 shows the forest plots comparing the sites of lesions seen on MRI between acute and chronic progressive NBD. Overall, there was no significant difference between the acute and chronic progressive groups regarding the following sites: brainstem (Figure 3A, OR = 1.19, *p* = 0.56), white matter (Figure 3B, OR = 0.92, *p* = 0.82), basal ganglia (Figure 3C; OR = 1.86, *p* = 0.17), and cerebellum (Figure 3D, OR = 1.11, *p* = 0.89), and spinal cord (Figure 3E, OR = 1.64, *p* = 0.46), and thalamus (Figure 3F, OR = 1.93, *p* = 0.21). Owing to the insignificant heterogeneity between the studies, the fixed model was used for all comparisons.

#### 3.7.3 MRI characteristics of parenchymal NBD

- Brainstem atrophy

**Figure 4:**
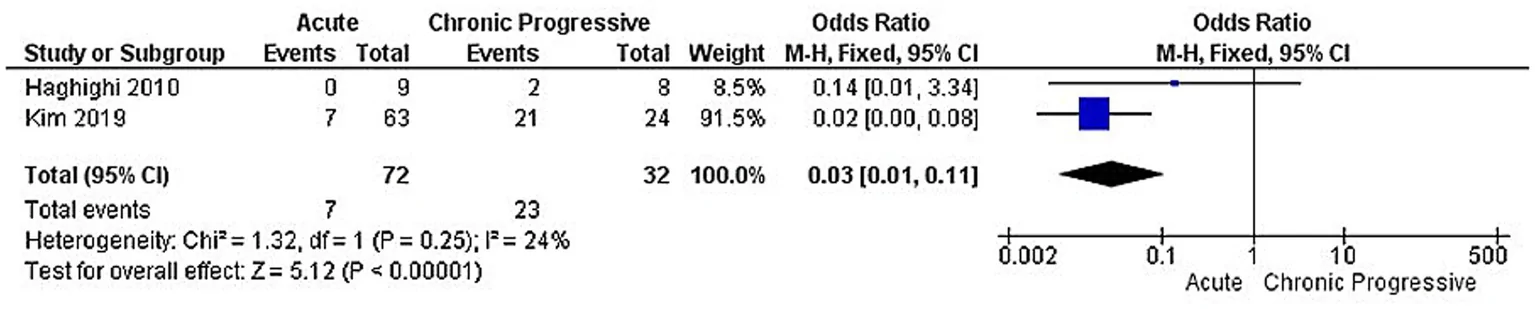
OR of brainstem atrophy between acute and chronic progressive NBD.

Figure 4 shows the incidence of brainstem atrophy was significantly lower in the acute NBD group than in the chronic progressive NBD group (Figure 4, OR = 0.03, 95% CI; [0.01, 0.11], *p* < 0.00001). The heterogeneity between the studies was insignificant so the Fixed model was used (*I*^2^ = 24%, *p* = 0.25).

- Enhancement lesion

**Figure 5:**
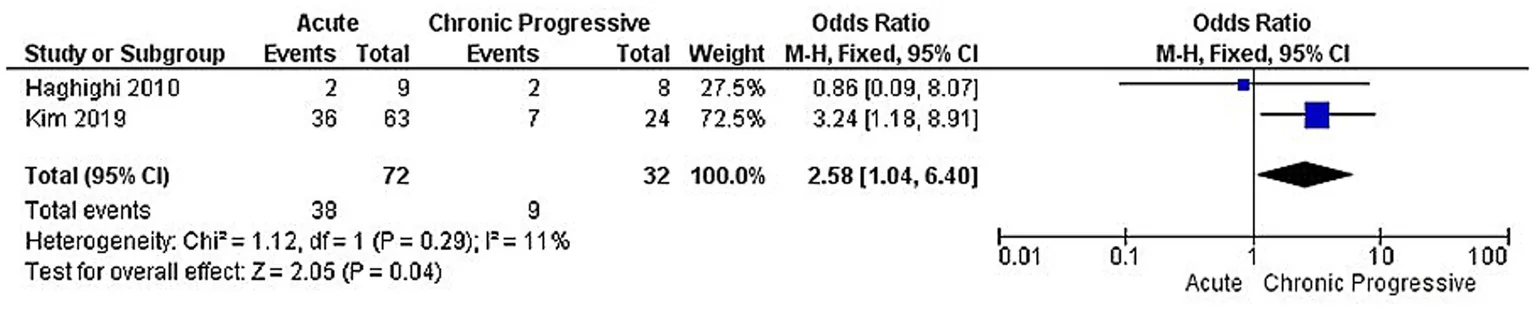
OR of enhancing lesion between acute and chronic progressive NBD.

Figure 5 shows the incidence of an enhancing lesion on MRI was significantly lower in the chronic progressive NBD group than in the acute NBD group (Figure 5, OR = 2.58, 95% CI; [1.04, 6.40], *p* = 0.04). The heterogeneity between the studies was insignificant; therefore, the fixed model was used (*I*^2^ = 11%, *p* = 0.29).

#### 3.7.4 MRI findings of NBD based on disease severity

**Figure 6:**
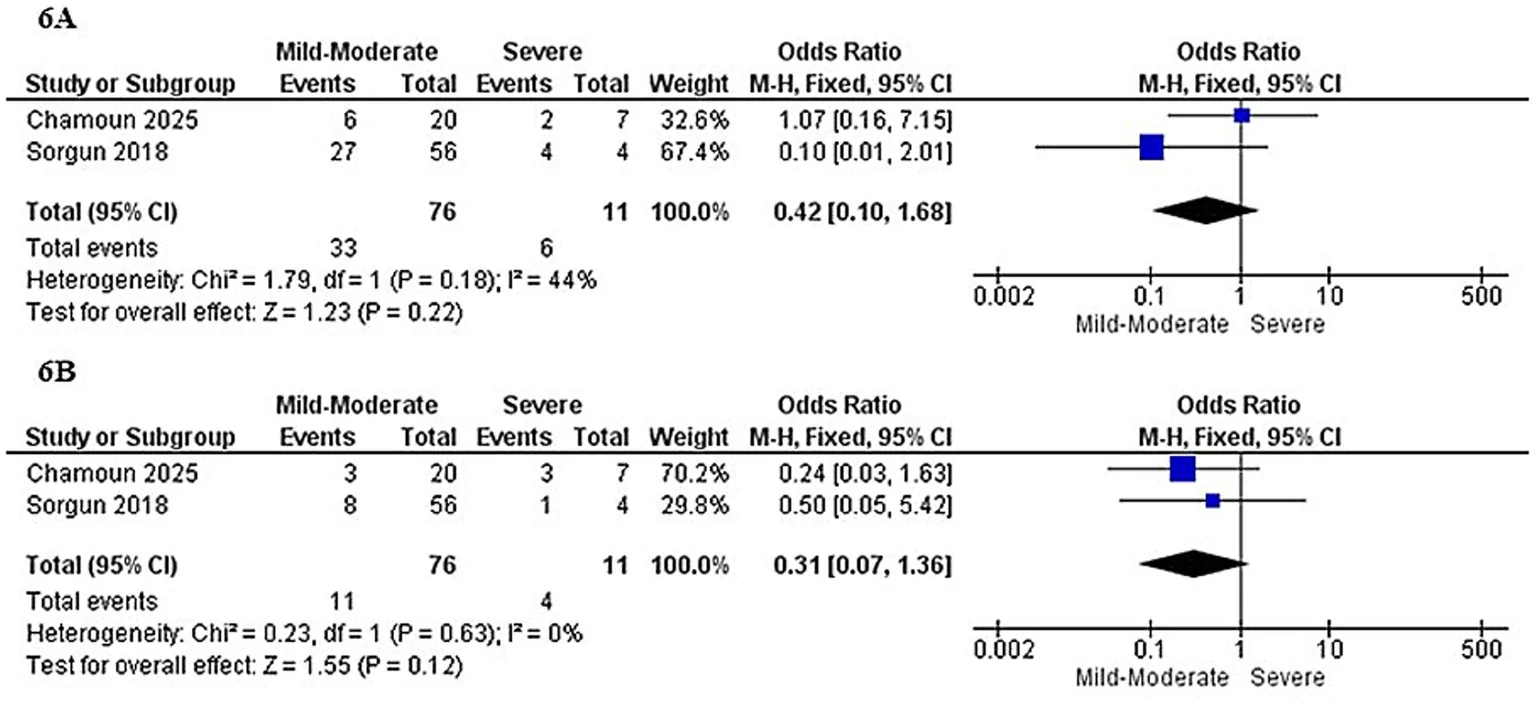
OR of site of lesions seen on MRI imaging between mild to moderate and severe NBD **(A)** Brainstem, **(B)** Thalamus.

Figure 6 shows the forest plots comparing the sites of lesions seen on MRI between mild-to-moderate NBD and severe NBD. Overall, there was no significant difference between the mild-to-moderate and severe groups regarding the following sites: brainstem (Figure 6A, OR = 0.42, *p* = 0.22), and thalamus (Figure 6B, OR = 0.31, *p* = 0.12).

## 4 Discussion

This systematic review and meta-analysis synthesized MRI findings from seven retrospective studies comprising 327 patients with parenchymal NBD. Our primary aim was to characterize the lesion topography and imaging features across clinical subtypes and disease severity.

### 4.1 MRI findings of parenchymal NBD

Our analysis did not reveal a statistically significant difference in the anatomical distribution of MRI lesions between acute and chronic progressive parenchymal NBD. This suggests that topography alone may not reliably distinguish between clinical phenotypes. Similarly, a systematic review by Al-Omoush et al. found that brainstem and diencephalic lesions are common in both acute and chronic presentations of the disease (7). Furthermore, Oumerzouk et al. reported in a large cohort that while acute lesions often appear in the mesodiencephalic junction and cerebral white matter, similar regions can also be involved in chronic progressive disease, although with different evolving imaging characteristics (9). A retrospective cohort study by Kim et al. further supports this, as they did not find a significant difference in the frequency of brainstem, thalamic, or basal ganglia lesions between the acute and chronic NBD groups (20). The consistency of lesion findings across subtypes is suggestive of shared neuroinflammatory pathways in NBD, possibly reflecting a common underlying vascular or autoimmune process targeting specific vulnerable neuroanatomical regions (8).

### 4.2 MRI characteristics of parenchymal NBD

Although lesion distribution did not differ meaningfully, our analysis identified two significant qualitative MRI features that distinguished acute from chronic progressive NBD. Brainstem atrophy was far more frequent in chronic progressive disease than in acute NBD (*p* < 0.00001). This is consistent with longitudinal studies suggesting that chronic progressive NBD is associated with progressive tissue loss due to sustained inflammation, particularly in the brainstem. Noet et al. observed that brainstem atrophy is a hallmark of long-standing progressive neurological involvement and is correlated with a poorer prognosis (23). This correlation is strongly supported by a meta-analysis by Ishido et al., which found a significant association between that brainstem atrophy was highly correlated with chronic progressive NBD than with acute NBD (76.3% vs. 11.9%, *p* < 0.001) (25). Second, contrast-enhancing lesions were significantly more common in acute NBD (*p* = 0.04), reflecting active blood–brain barrier disruption and inflammation. Peine et al. also identified contrast enhancement as a key radiological feature associated with acute disease activity, reflecting a potential threshold for treatment escalation (26). The presence of enhancement on MRI may potentially serve as an imaging biomarker for disease activity, while atrophy could indicate irreversible neurological damage as part of a progressive clinical course, but future prospective studies are required to validate these associations. Taken together, these findings suggest that qualitative MRI features—particularly brainstem atrophy and contrast enhancement—are more informative than lesion topography alone for distinguishing acute from chronic progressive parenchymal NBD and may have important implications for prognosis and long-term disease management. These results suggest that future MRI classification systems should incorporate quantitative measures of atrophy and enhancement patterns rather than relying solely on anatomical sites.

### 4.3 MRI findings based on disease severity

Our comparison of MRI lesion locations between mild-to-moderate and severe NBD groups revealed no significant differences in brainstem or thalamic involvement. This suggests that disease severity may not be directly inferred from the presence or location of lesions but rather from their extent or associated complications, such as atrophy or enhancement. This is supported by the findings of Chaomen et al.’s study, which found that disability in NBD correlated strongly with brainstem atrophy and diffuse white matter involvement rather than isolated lesion counts (11). Sorgun et al. did not find any significant difference in anatomical site involvement between mild-to-moderate and severe NBD, although they identified that hemispheric involvement is far more common in severe NBD, albeit in a small sample size (19).

### 4.4 Resolution of brain lesions in MRI follow-up against persistent lesions

The limited data in the included studies hindered a meta-analysis of lesion evolution. However, the existing literature on brain lesion follow-up on MRI is scarce yet shares some similar outcomes. Ayoade et al. found ring-enhancing lesions of the right thalamus, midbrain, and pons on follow-up MRI in a single patient treated for acute NBD (27). In contrast, chronic progressive NBD is associated with persistent T2 lesions and progressive brainstem or cerebral atrophy. The presence of persistent lesions or atrophy on follow-up MRI has been linked to worse long-term neurological outcomes and may suggest the need for more aggressive immunomodulation.

### 4.5 Nonparenchymal lesions of NBD

Our review primarily focused on parenchymal NBD. However, the literature highlights that nonparenchymal NBD presents with distinct MRI features, including venous sinus thrombosis, hemorrhagic infarction, and diffuse cerebral edema (7). MRI with venography is essential for the diagnosis of nonparenchymal NBDs with imaging characteristics such as cerebral venous thrombosis. Unlike parenchymal lesions, cerebral venous thrombosis in NBD often responds well to anticoagulation and corticosteroids (5, 7), although residual neurological deficits may occur if the diagnosis is delayed. Recognizing this dichotomy is critical for an accurate diagnosis.

### 4.6 Clinical implications

Our findings reinforce that MRI remains indispensable for the evaluation of NBD subtyping and assessment. Radiologists and neurologists should carefully evaluate qualitative MRI features, such as contrast enhancement and signs of atrophy, as these findings may provide valuable information regarding disease phenotype and activity. These imaging findings may have some considerable implications for treatment; enhancing lesions may require more aggressive immunosuppression, whereas atrophy may indicate irreversible damage that guides rehabilitation planning. Although enhancing lesions may reflect inflammation and brainstem atrophy that indicate chronic neurological damage, their role as imaging biomarkers for prognosis and management plan formation requires confirmation in larger prospective studies. Therefore, standardized MRI acquisition protocols and reporting criteria for NBD are needed to improve consistency across future studies and facilitate validation of these imaging characteristics.

### 4.7 Limitations

This study had several limitations. First, all the included studies were retrospective, introducing potential selection and confounding bias. Information about immunosuppressive regimens and timing of therapy initiation was inconsistently reported, precluding quantitative evaluation of their influence on the MRI results. Consequently, the associations identified in our review should be interpreted independently of treatment affects. Also, patients with delayed diagnosis and untreated disease may be more likely to demonstrate chronic structural abnormalities on imaging, yet the included studies seldom reported the interval between symptom onset and MRI acquisition. Several potential confounders were not completely reported across the included studies such as comorbid conditions, systemic disease activity, and other baseline clinical characteristics, which could potentially account for the variability among the observed studies. Second, heterogeneity in MRI acquisition protocols and reporting methods may have affected the detection and characterization of lesions in the reported studies. Differences in imaging sequences, use of contrast, and radiological interpretation could have contributed to heterogeneity in the reported MRI results, thereby limiting the comparability of pooled imaging outcomes. Third, the number of included studies (*n* = 7) limited the statistical power, particularly comparisons of severity. Fourth, we could not analyze longitudinal lesion evolution because of insufficient follow-up data in the included studies. Finally, our focus on parenchymal NBD excluded insights from nonparenchymal cases, which represent a clinically significant subgroup.

## 5 Conclusion

Our findings suggest demonstrate that while the anatomical distribution of lesions does not significantly differ between acute and chronic progressive subtypes or between mild-to-moderate and severe disease states, specific qualitative MRI features serve as critical discriminators. Brainstem atrophy appears to be associated with chronic progressive disease, potentially reflecting irreversible neurological damage; however, contrast-enhancing lesions are strongly associated with acute inflammatory activity. These findings suggest that qualitative imaging characteristics may be more informative than lesion topography alone for distinguishing acute from chronic parenchymal NBD, although this interpretation should be explored in larger prospective studies. Future studies are essential to validate these findings and clarify longitudinal lesion evolution for patients with this complex neurological disorder.

## Data Availability

The original contributions presented in the study are included in the article/Supplementary material, further inquiries can be directed to the corresponding author.
