# Supplementary material for: MRI features of parenchymal neuro-Behçet’s disease: a systematic review and meta-analysis
Source: Front Neurol. 2026 Aug 19;17:1818678. doi: 10.3389/fneur.2026.1818678 (PMC13533666; doi:10.3389/fneur.2026.1818678)
Supplement: Supplementary file 1 [file Table_1.DOCX]

Supplementary Material

Table 1. Search strategy

("Behcet Syndrome"[Mesh] OR Behcet*[tiab] OR Behçet*[tiab] OR "neuro-Behcet*"[tiab] OR "neuro Behcet*"[tiab]) AND ("Magnetic Resonance Imaging"[Mesh] OR MRI[tiab] OR "magnetic resonance" [tiab] OR "MR imaging"[tiab] OR "diffusion-weighted"[tiab] OR DWI[tiab] OR FLAIR[tiab] OR SWI[tiab] OR "contrast-enhanced"[tiab]) AND (brain[tiab] OR brainstem[tiab] OR midbrain[tiab] OR thalamus[tiab] OR "basal ganglia"[tiab] OR cerebellum *[tiab] OR "internal capsule"[tiab] OR "cerebral peduncle*"[tiab] OR "spinal cord"[tiab] OR venous[tiab] OR sinus[tiab] OR "venous sinus thrombosis"[tiab] OR CVST[tiab] OR dural[tiab] OR aneurysm*[tiab] OR vascular *[tiab]) NOT (animals[mh] NOT humans[mh]).

Table 2. Clinical characteristics of parenchymal NBD

| Study ID | Characteristics of BD | | | | | | | | | | | | Neurological characteristics | | | | | | | | | | | | | | | | | | | |
| --- | --- | --- | --- | --- | --- | --- | --- | --- | --- | --- | --- | --- | --- | --- | --- | --- | --- | --- | --- | --- | --- | --- | --- | --- | --- | --- | --- | --- | --- | --- | --- | --- |
|  | **Oral aphthous ulcers** | | **Genital ulcerations** | | **Ocular manifestations** | | **Joint involvement** | | **Venous thrombosis** | | **Arterial involvement** | | **Headaches** | | **Hemi/paraparesis** | | **Sensory symptoms** | | **Cranial nerve involvement** | | **Ataxia** | | **Sphincter dysfunction** | | **Confusion** | | **Coma** | | **Seizures** | | **Optic neuropathy** | |
|  | **A-NB** | **CP-NB** | **A-NB** | **CP-NB** | **A-NB** | **CP-NB** | **A-NB** | **CP-NB** | **A-NB** | **CP-NB** | **A-NB** | **CP-NB** | **A-NB** | **CP-NB** | **A-NB** | **CP-NB** | **A-NB** | **CP-NB** | **A-NB** | **CP-NB** | **A-NB** | **CP-NB** | **A-NB** | **CP-NB** | **A-NB** | **CP-NB** | **A-NB** | **CP-NB** | **A-NB** | **CP-NB** | **A-NB** | **CP-NB** |
| Kim  2019 )21( | **97 (99.0)** | | **66 (67.3)** | | **57 (58.2)** | | ***N/A*** | | ***N/A*** | | ***N/A*** | | **45 (45.9)** | | ***N/A*** | | ***N/A*** | | ***N/A*** | | **22 (22.4)** | | ***N/A*** | | **(10.2%)** | | ***N/A*** | | **(9.2%)** | | **3 (3.1)** | |
| Haghighi 2010 )22( | **N/A** | | **12 (71)** | | **8 (47)** | | **6 (35)** | | **5 (29)** | | | | ***N/A*** | | | | | | | | | | | | | | | | | | | |
| Demir 2003 )23( | ***N/A*** | | | | | | | | | | | | | | | | | | | | | | | | | | | | | | | |
| Noel  2014 )24( | **78 (100)** | **37 (100)** | **56 (72)** | **23 (62)** | **54  (69)** | **20  (54)** | **36 (46)** | **16 (43)** | **27 (35)** | **7 (19)** | **8  (10)** | **4  (11)** | **51 (65)** | **20 (54)** | **44  (56)** | **15  (41)** | **19 (24)** | **11 (30)** | **21 (27)** | **7  (19)** | **17 (22)** | **13 (35)** | **11 (14)** | **10 (27)** | **4 (5)** | **7 (19)** | **6 (8)** | **1 (3)** | **2 (3)** | **0 (0)** | **1  (1)** | **0  (0)** |
| Ideguchi 2010 )25( | **54  (100)** | | **33  (61)** | | **42  (78)** | | **20  (37)** | | **2  (4)** | | | | **25 (66)** | **3 (20)** | ***N/A*** | ***N/A*** | **10 (26)** | **4 (27)** | **10 (26)** | **6  (40)** | **4 (11)** | **6 (40)** | **4  (11)** | **8 (53)** | ***N/A*** | ***N/A*** | ***N/A*** | ***N/A*** | ***N/A*** | ***N/A*** | ***N/A*** | ***N/A*** |

**Abbreviations**: BD; Behçet's Disease, A-NB; Acute Neuro-Behçet's Disease, CP-NB; Chronic Progressive Neuro-Behçet's Disease.

Table 3. Baseline characteristics, clinical manifestations of NBD based on disease severity

| Study ID | Total | Baseline characteristics | | | | | | | | | | | | | | Non-neurologic symptoms, n (%) | | | | | | | | | | | | | |
| --- | --- | --- | --- | --- | --- | --- | --- | --- | --- | --- | --- | --- | --- | --- | --- | --- | --- | --- | --- | --- | --- | --- | --- | --- | --- | --- | --- | --- | --- |
|  |  | **Number of patients, n** | | **Age, year, (mean ± SD)** | | **Female,  n (%)** | | **Male,  n (%)** | | **Onset age of BD (years)** | | **Age at onset of NBD** | | **HLA B51** | | **Oral ulcers** | | **Genital ulcers** | | **Uveitis** | | **GI involvement** | | **Arthritis or arthralgia** | | **Cutaneous involvement** | | **DVT** | |
|  |  | M-NBD | S-NBD | M-NBD | S-NBD | M-NBD | S-NBD | M-NBD | S-NBD | M-NBD | S-NBD | M-NBD | S-NBD | M-NBD | S-NBD | M-NBD | S-NBD | M-NBD | S-NBD | M-NBD | S-NBD | M-NBD | S-NBD | M-NBD | S-NBD | M-NBD | S-NBD | M-NBD | S-NBD |
| Sorgun 2018 )19( | 60 | 56 | 4 | 44.8±11.7 | 53.8±9.1 | 22 (39.3) | 1  (25) | 34 (60.7) | 3 (75) | 30.18±10.3 | 32.52±6.55 | 37.5±10.9 | 50.25±9.1 | 3 (5.4) | N/A | 56 (100) | 4 (100) | 42 (75) | 4 (100) | 31 (55.4) | 3  (75) | 6 (10.7) | 0 (0) | 28 (50) | 1  (25) | 26 (46.4) | 1  (25) | 10 (17.9) | 0 (0) |
| Chamoun 2025 )20( | 27 | 20 | 7 | 46 ± 14.6 | 51.14 ± 9.8 | 9 (45.0) | 5 (71.4) | N/A | N/A | 31.3 ± 13.4 | 35.3 ± 13.7 | 37.4 ± 14.5 | 39.8 ± 11.5 | N/A | N/A | 17 (85.0) | 6 (85.7) | 15 (75.0) | 4 (57.1) | 7 (35.0) | 5 (71.4) | 6 (30.0) | 0 (0) | 10 (50.0) | 4 (57.1) | 8 (40.0) | 4 (57.1) | 2 (10.0) | 1 (14.3) |

**Abbreviations**: M-BD; Mild to Moderate Behçet's Disease, S-BD; Sever Behçet's Disease, GI; Gastrointestinal, DVT; Deep Vein Thrombosis.

Table 4. Neurological manifestations of NBD based on disease severity

| Study ID | Neurologic symptoms, n (%) | | | | | | | | | | | |
| --- | --- | --- | --- | --- | --- | --- | --- | --- | --- | --- | --- | --- |
|  | **Headache** | | **Dizziness** | | **Weakness** | | **Diplopia** | | **Dysphagia** | | **Cognitive/behavioral changes** | |
|  | M-NBD | S-NBD | M-NBD | S-NBD | M-NBD | S-NBD | M-NBD | S-NBD | M-NBD | S-NBD | M-NBD | S-NBD |
| Sorgun 2018 )19( | N/A | | | | | | | | | | | |
| Chamoun 2025 )20( | 19 (95.0) | 5 (71.4) | 8 (40.0) | 3 (42.9) | 13 (65.0) | 4 (57.1) | 2 (10.0) | 2 (28.6) | 1 (5.0) | 0(0) | 4 (20.0) | 3 (42.9) |

**Abbreviations**: M-BD; Mild to Moderate Behçet's Disease, S-BD; Sever Behçet's Disease

Table 5. Newcastle-Ottawa Scale for the included cohort retrospective studies

|  | **Selection** | | | | **Comparability** | **Outcome** | | | **Quality Score** | **Risk of Bias (0-3:High, 4-6: Moderate, 7-9: Low)** |
| --- | --- | --- | --- | --- | --- | --- | --- | --- | --- | --- |
| Article | **Q1** | **Q2** | **Q3** | **Q4** | **Q5** | **Q6** | **Q7** | **Q8** |  |  |
| Sorgun, 2018 | * | No non-exposed comparator. | * | Disability measured only at last visit. | No adjustment for confounders. | * | N/A | N/A | 3 | High |
| Chamoun, 2025 | * | * | * | Baseline outcome status not confirmed. | No adjustment for confounders. | * | * | * | 6 | Moderate |
| Kim, 2019 | * | No BD-without-NBD group included. | * | Outcome already present | No adjustment for confounders | * | * | N/A | 4 | Moderate |
| Haghighi, 2010 | * | No non-exposed comparator. | * | Baseline MRI already showed lesions in most patients. | No adjustment for confounders | * | * | * | 5 | Moderate |
| Demir, 2003 | * | No non-exposed comparator. | * | MRI findings and neurological status already present at inclusion. | No adjustment for confounders | * | * | Incomplete follow-up | 4 | Moderate |
| Noel, 2014 | * | No non-exposed comparator. | * | MRI findings and neurological status already present at inclusion. | * | * | * | N/A | 5 | Moderate |
| Ideguchi, 2010 | * | * | * | MRI findings and neurological status already present at inclusion. | No adjustment for confounders | * | * | N/A | 5 | Moderate |
| **Selection:** Q1. Representativeness of the exposure cohort? Q2. Selection of the non-exposure cohort? Q3. Ascertainment of exposure? Q4. Demonstration that outcome of interest was not present at start of the study?  **Comparability:** Q5. Comparability of cohort on the basis of the design or analysis?  **Outcome: Q6**. Assessment of outcome? Q7. Was follow-up long enough for outcomes to occur? Q8. Adequacy of follow-up of cohorts? | | | | | | | | | |  |
